# Myristoylated Cathelicidin-DM Fused With ANG1-7: A Novel Self-Assembling Antimicrobial Peptide for the Treatment and Mechanism of Diabetic Infected Wounds

**DOI:** 10.1155/jdr/9601959

**Published:** 2025-09-02

**Authors:** Rongqin Feng, Peng Wang, Li Fan, He Lu, Danna Yao, Panpan Sun, Zhonghua Liu, Fu Han, Xiaozhi Bai, Xuekang Yang, Juntao Han

**Affiliations:** ^1^College of Life Sciences, Northwest University, Xi'an, Shaanxi, China; ^2^Department of Burns and Cutaneous Surgery, Xijing Hospital, The Fourth Military Medical University, Xi'an, Shaanxi, China

**Keywords:** antibacterial, anti-biofilm, antimicrobial peptide, diabetic wound healing, infection, self-assembly

## Abstract

Diabetic wounds, due to severe vascular dysfunction, persistent inflammatory responses, and susceptibility to microbial infections, exhibit delayed healing and pose a significant challenge to human health. Diabetic wounds face delayed healing and significant health challenges due to vascular dysfunction, persistent inflammation, and infection susceptibility. Therefore, the development of drugs with antibacterial capabilities, as well as the ability to effectively regulate inflammation and promote angiogenesis, is of great importance. In this study, a novel antibacterial peptide (named MYR-DM-ANG1-7) was designed. It is composed of the coassembly of myristoylated antibacterial peptide cathelicidin-DM and angiotensin 1-7 (ANG 1-7). This novel antibacterial peptide demonstrates antibacterial activity against both *Escherichia coli* and *Staphylococcus aureus* bacteria and can even effectively inhibit the formation of biofilms. In vitro experiments confirmed that MYR-DM-ANG1-7 can promote the proliferation, migration, and angiogenesis of human umbilical vein endothelial cells (HUVECs), reduce the level of oxidative stress, alleviate the increase in mitochondrial membrane potential caused by high glucose (HG) and lipopolysaccharide (LPS), and decrease the expression of proinflammatory cytokines IL-6 and TNF-*α*. Western blot experiments confirmed that MYR-DM-ANG1-7 activates PI3K by targeting the membrane receptor Mas, thereby activating AKT, which ultimately promotes the activation of eNOS to produce nitric oxide (NO), thereby enhancing the angiogenic capacity of HUVECs. In vivo experiments showed that the local application of MYR-DM-ANG1-7 significantly improved the healing of infected diabetic wounds in mice, including increased wound healing rate, reduced inflammatory cell infiltration, and promoted collagen fiber and blood vessel formation. In summary, this study successfully constructed a multifunctional novel self-assembling antibacterial peptide that can effectively regulate oxidative stress, inflammation, and angiogenesis to promote the repair of diabetic infected wounds. This research provides a brand new self-assembling lipopeptide therapeutic strategy for the treatment of diabetic infected wounds.

## 1. Introduction

Diabetic foot ulcers (DFUs) remain a major clinical challenge. Recent data from the International Diabetes Federation (IDF) indicate that 19%–34% of diabetic wounds progress to chronic ulcers, with patients exhibiting concurrent bacterial infection facing a significantly increased risk of amputation compared to those without infections [1–3]. The synergistic interplay between pathogenic biofilm formation and the hyperglycemic microenvironment underlies the impairment of vascular regeneration [4, 5]. On the one hand, elevated glucose levels induce the accumulation of advanced glycation end products (AGEs) in vascular endothelial cells, which antagonize VEGF/FGF-mediated proangiogenic signaling. Simultaneously, AGEs activate the RAGE-NF-*κ*B pathway, downregulating endothelial nitric oxide synthase (eNOS) expression and reducing nitric oxide (NO) bioavailability, thereby markedly impairing endothelial migration [6–8]. On the other hand, pathogenic bacteria within infected wounds secrete effector proteins such as elastase and *α*-toxin, which disrupt interendothelial junctional proteins. Concurrently, hyperactivation of matrix metalloproteinase-9 (MMP-9) in diabetic wound microenvironments exacerbates the degradation of Type IV collagen and laminin. These synergistic mechanisms collectively accelerate the breakdown of the basement membrane underlying nascent vasculature in the wound bed. Thus, the development of novel dual-target therapies capable of eliminating pathogenic biofilms while concurrently restoring a provasculogenic microenvironment has emerged as a pivotal strategy to address the therapeutic bottleneck in diabetic wound management.

The rising prevalence of multidrug-resistant bacterial infections in chronic wound management has highlighted the limitations of conventional antibiotics, driving the exploration of novel antimicrobial agents. Antimicrobial peptides (AMPs), as innate immune defense molecules, exhibit broad-spectrum antimicrobial activity and low resistance induction potential due to their dual mechanisms of membrane disruption (e.g., pore formation) and intracellular targeting [9, 10]. However, substantial heterogeneity in antimicrobial efficacy exists among AMPs from different species, primarily attributed to variations in hydrophobicity and charge profiles. Among these [11], amphibian-derived AMPs are regarded as promising candidates for therapeutic development due to their multifunctional bioactivities, including broad-spectrum antibacterial effects (against both Gram-positive and Gram-negative bacteria), antibiofilm properties, host cell proliferation promotion, and immunomodulatory functions [12–14].

Through targeted screening of peptide databases against common diabetic wound pathogens, a novel *α*-helical AMP cathelicidin-DM (DM) derived from amphibians was identified. In vivo studies demonstrated that systemic administration of DM significantly accelerated wound healing in rodent models with infected wounds, accompanied by enhanced vascular regeneration. In vitro investigations further revealed DM's dual functionality: direct inhibition of *Staphylococcus aureus* and *Escherichia coli*, as well as promotion of human umbilical vein endothelial cell (HUVEC) proliferation and tube formation. These findings suggest DM's potential as a dual therapeutic agent for diabetic wound infection and vascular impairment.

Despite their in vitro efficacy, the clinical translation of natural AMPs is hindered by challenges in the wound microenvironment [15, 16], including proteolytic degradation, immune clearance (e.g., by macrophages or neutrophils), and charge shielding effects induced by ionic components. To address these limitations, DM was structurally optimized using a two-step modification strategy. First, myristic acid (C14) was conjugated to the N-terminus via lipidation, generating MYR-DM. This modification preserved the inherent *α*-helical conformation while enabling supramolecular self-assembly into micellar nanostructures through hydrophobic interactions and hydrogen bonding [17]. These nanoscale assemblies enhanced localized drug retention and potentiated antibacterial activity by disrupting bacterial membrane integrity [18–20]. Prior research by Lei et al. demonstrated that myristic acid conjugation at the N-terminus of HBD5 similarly enhanced antimicrobial efficacy through analogous supramolecular self-assembly mechanisms [21].

ANG1-7, an endogenous bioactive heptapeptide and ligand for the Mas receptor (MasR) [22], exerts vascular protective effects by antagonizing ANG II/AT1R signaling and activating the PI3K/AKT/eNOS pathway to promote NO release [23, 24]. In diabetic wounds, research found that ANG1-7 may ameliorate angiogenesis impairment, as evidenced by studies showing its capacity to mitigate hyperglycemia-induced podocyte injury [25] and suppress JAK2/STAT3-mediated endothelial inflammation [24]. To synergistically address diabetic vascular dysfunction, ANG1-7 (DRVYIH) was covalently linked to the C-terminus of MYR-DM via a GGG flexible spacer. Structural analysis confirmed that ANG1-7 integration increased the peptide's net positive charge, potentially enhancing electrostatic interactions with negatively charged bacterial membranes.

The engineered amphiphilic peptide, MYR-DM-ANG1-7, combines dual therapeutic modules: the N-terminal myristoyl group facilitates self-assembly into nanoscale structures, promoting localized accumulation and broad-spectrum antimicrobial activity against pathogens (including drug-resistant strains and biofilms) such as *S. aureus* and *E. coli.* In a diabetic murine full-thickness skin defect model, topical application of MYR-DM-ANG1-7 markedly improved wound closure rates and increased neovascularization density. Concurrently, in vitro experiments under hyperglycemic conditions demonstrated that the C-terminal ANG1-7 moiety targets vascular endothelial MasR, reversing microvascular dysfunction via the PI3K/AKT/eNOS pathway activation. This integrative design strategy, merging antimicrobial and proangiogenic functions within a single molecule, offers a novel therapeutic paradigm for diabetic wounds complicated by resistant infections and microcirculatory impairment.

## 2. Materials and Methods

### 2.1. Preparation of Self-Assembling Peptides

The AMP cathelicidin-DM was synthesized by Qiangyao Biotechnology (Shanghai, China). The AMP MYR-DM-ANG1-7 was synthesized by Hangzhou Gutuo Biotechnology Co., Ltd. (Hangzhou, China) using the standard solid-phase 9-fluorenylmethoxycarbonyl (Fmoc) method. The peptides were purified to > 95% using reverse-phase high-performance liquid chromatography (HPLC) and were verified using a mass spectrometer. The final products were lyophilized and stored at −80°C until use.

### 2.2. Bacterial Strains, Growth Conditions, and Reagents

All tested bacterial strains were obtained from the laboratory of the Department of Clinical Laboratory at the First Affiliated Hospital of Air Force Medical University. The cultures of all bacterial strains were grown on blood agar plates at 37°C.

### 2.3. Transmission Electron Microscopy (TEM) of Self-Assembled Structures

The self-assembly morphology of peptide amphiphiles in water was observed using TEM. Each sample was mounted on a 300-mesh copper-coated carbon grid and then negatively stained with a 1.5% phosphotungstic acid solution (1.5% PTA). Before imaging, all samples were air-dried for approximately 10 min. Finally, the samples were characterized under an acceleration voltage of 80 kV.

### 2.4. Assessment of Bacterial Growth Inhibition

The minimum inhibitory concentrations (MICs) of DM and MYR-DM-ANG1-7 were determined using the broth microdilution method. *E. coli* (ATCC 25922) and *S. aureus* (ATCC 25923) were cultured overnight in Mueller–Hinton broth (MHB) at 37°C. The cultures were diluted to 2 × 10^5^ CFU/mL. The peptides were serially diluted in 10 mM phosphate-buffered solution (PBS) and added to a 96-well plate. The bacterial suspensions were mixed with an equal volume of peptide solution and incubated at 37°C for 18 h. Bacterial growth inhibition was determined by measuring the absorbance at 600 nm using a M200 multiwavelength microplate reader (Infinite M200 PRO, Switzerland). Subsequently, bacterial suspensions were treated with 1 × MIC, 2 × MIC, 3 × MIC, and 4 × MIC concentrations of the AMPs and sampled at 0, 3, 6, 12, 18, and 24 h. The absorbance was measured at 600 nm. The experiment was repeated three times.

### 2.5. Antibiofilm Activity Experiment

*E. coli* (ATCC 25922) and *S. aureus* (ATCC 25923) were cultured overnight in MHB medium at 37°C. The bacterial suspension was diluted to 100 times and added to a 12-well plate (500 *μ*L/well). Each peptide with a different concentration was added to the corresponding well plate and incubated at 37°C for 24 h. After incubation, 100 *μ*L of culture supernatant was discarded. The biofilm was fixed with 100 *μ*L of 100% methanol for 15 min, then air-dried, then dyed with 0.1% crystal violet (CV) for 30 mins, and washed with distilled water three times. After adding 200 *μ*L of 95% ethanol, the inhibition rate of the biofilm was calculated by measuring the OD value at 595 nm with Infinite M200 PRO, Switzerland. After ultrasonic treatment, the biofilm was fully dispersed into single cells, and the cell suspension was diluted to a suitable concentration for colony counting. All experiments were repeated three times.

### 2.6. Hemolysis Test

In order to evaluate the hemolytic activity of self-assembled peptides on normal mammalian cells, fresh blood of normal mice was collected, and heparin sodium was anticoagulated and stored. After centrifugation at 4°C and 3000 rpm for 10 min, the precipitate was collected, washed, and centrifuged with normal saline for three times and then resuspended at 10% (*v*/*v*). We mix 100 *μ*L of different concentrations of drugs with 100 *μ*L of mouse red blood cell suspension, incubate in a constant temperature oscillation incubator at 37°C for 60 min, then take out and centrifuge according to the above setting, absorb 100 *μ*L of supernatant, put it in a 96-well cell culture plate, and measure the OD value at 590 nm by M200 full-band enzyme-labeled instrument (Infinite M200 Pro, Switzerland). Then, 100 *μ*L of 0.1% Triton X-100 solution was used as a positive control and PBS as a negative control. According to the formula, hemolysis (%) = (experimental group OD value − negative control OD value)/(positive control OD value − negative control OD value) × 100%.

### 2.7. Analysis of Cell Viability

Human keratinocyte cells (HaCaTs) and human skin fibroblasts (HSFs) were seeded in 96-well plates at a density of 5000 cells per well. After overnight culture, different concentrations of AMP MYR-DM-ANG1-7 were added to the cells, and then, the cells were cultured for 24 h. Untreated cells were supplemented with fresh culture medium as the control group. Subsequently, 10% by volume of CCK8 solution (5 mg/mL) was added to each well.

After incubation for 1 h, the absorbance in each well was measured at 450 nm using an M200 full-band microplate reader (Infinite M200 PRO, Switzerland). Finally, the cell viability was calculated relative to the control groups.

### 2.8. Cell Culture

HUVECs were provided by Wuhan Sevier Biotechnology Co., Ltd. HUVECs were cultured in DMEM containing 5% CO_2_, 37°C, and 95% air.

### 2.9. Cell Proliferation Experiment

HUVECs were seeded into a 96-well plate at a density of 5000 cells per well. After overnight incubation, different concentrations of drugs were added to the cells and then incubated for 24 h. Untreated cells supplemented with fresh medium were used as controls. Subsequently, a 10-*μ*Lvolume of CCK-8 solution (5 mg/mL) was added. After incubation for 1 h, the absorbance in each well was measured at 450 nm using an M200 full-band microplate reader (Infinite M200 PRO, Switzerland). Finally, the average optical density (OD) of the three wells in the indicated groups was used to calculate the percentage of cell viability according to the following formula: Cell viability (%) = (OD treated group − OD blank group)/(OD control group − OD blank group) × 100%.

### 2.10. Transwell Detection and Scratch Wound Healing Detection

HUVECs (1 × 10^4^ cells/well) were incubated in the upper chamber of an 8-*μ*m pore size (Corning, New York) and placed into a 24-well plate for 16 h at 37°C with 5% CO_2_. Cells adhering to the top surface of the Transwell membrane were carefully removed using a cotton swab. Cells that had migrated to the bottom of the chamber were fixed with 4% paraformaldehyde for 30 min and stained with 0.1% CV for 20 min. Finally, the chambers were placed on slides and imaged using an inverted fluorescence microscope (ThermoFish EVOS, United States). The number of cells in five random fields of each chamber was counted to determine migration ability.

In a 6-well plate, HUVECs (2 × 10^5^ cells/well) were seeded. The cells were starved in serum-free medium for 12 h, and then, a scratch was made vertically using a 200-*μ*L pipette tip. Finally, the cells were imaged at 0, 12, and 24 h using an inverted fluorescence microscope (ThermoFish EVOS, United States).

### 2.11. Angiogenesis Experiment

The formation of vascular structure was observed by matrix glue experiment, and the angiogenesis ability was evaluated in Matrigel stent (Corning, New York). After treatment in each group, 300 *μ*L (2 × 10^5^ cells/mL) HUVECs were digested and inoculated into a 24-well plate containing Matrigel scaffold. After 9 h, the formation image of the tube was photographed.

### 2.12. Measurement of the Mitochondrial Membrane Potential (MMP)

JC-1 is a widely used and ideal fluorescent probe for detecting MMP. When the MMP is high, JC-1 accumulates in the matrix of the mitochondria, forming J-aggregates that emit red fluorescence. When the MMP is low, JC-1 cannot accumulate in the mitochondrial matrix, remaining as a monomer that emits green fluorescence. HUVECs were cultured in well plates containing DMEM at a density of 1 × 10^6^ cells/well. Following the indicated treatments, the slides were washed three times with PBS and the cells were incubated with 1 mg/l JC-1 at 37°C for 30 min in the incubator, washed briefly three times with PBS. The fluorescence was measured over the entire field of vision using a fluorescent microscope connected to an inverted fluorescence microscope (ThermoFish EVOS, United States). The experiment was repeated three times.

### 2.13. Measurement of Reactive Oxygen Species

To evaluate the antioxidant activity of MYR-DM-ANG1-7, HUVECs were cultured in DMEM supplemented with 10% FBS at 37°C and 5% CO_2_. After serum starvation for 24 h, the cells were divided into five experimental groups: normal control group, high glucose + LPS, high glucose + DM group, and high glucose + MYR-DM-ANG1-7. After drug treatment, the cells were washed three times with ice-cold PBS and incubated with 10 *μ*M DCFH-DA probe in serum-free medium at 37°C in the dark for 20 min. After removing the probe with PBS, the fluorescence intensity of 10,000 live cells was immediately measured by flow cytometry (excitation: 488 nm and emission: 530/30 nm). The data were expressed as the geometric mean fluorescence intensity (MFI) of each sample.

### 2.14. Enzyme-Linked Immunosorbent Assay (ELISA) for the Detection of Interleukin IL-6 and Tumor Necrosis Factor (TNF)-*α* in Culture Supernatant

The HUVECs were seeded at 1 × 10^4^ cells/well and cultured in 96-well plates. Following the indicated treatments, the levels of IL-6 and TNF-*α* in culture media were analyzed using ELISA according to the method of the kit. The experiments were repeated three times.

### 2.15. Measurement of Malondialdehyde (MDA) Activity

The level of MDA was determined by the thiobarbituric acid (TBA) method. Briefly, 100 *μ*L of cell supernatant was mixed with 200 *μ*L of a 10% trichloroacetic acid solution of 0.67% TBA. The mixture was incubated at 95°C for 30 min, cooled on ice, and centrifuged at 12,000 × *g* at 4°C for 10 min. The absorbance of the supernatant was measured at 532 nm using an enzyme-labeled instrument. The experiments were repeated three times.

### 2.16. Measurement of NO Activity

NO levels were determined using the Griess reaction. Briefly, 100 *μ*L of cell culture supernatant was mixed with an equal volume of Griess reagent and incubated at room temperature in the dark for 30 min. The absorbance of the reaction mixture was measured at 540 nm using a microplate reader. Sodium nitrite (NaNO_2_) was used as a standard to generate a calibration curve, and the NO concentration in the samples was calculated accordingly. All experiments were independently performed in triplicate to ensure data reliability and reproducibility.

### 2.17. Western Blot Analysis

After the specified treatments, HUVECs were harvested using a cell scraper and lysed in cell lysis buffer (Wuhan Boster Biological Engineering Co., Ltd.) for 30 min at 4°C. Total protein was quantified using a BCA protein assay kit. Lysate (20 *μ*g) from each sample was separated by 10% sodium dodecyl sulfate–polyacrylamide gel electrophoresis (SDS-PAGE) and then transferred to a polyvinylidene fluoride (PVDF) membrane. The membrane was blocked with a fresh blocking buffer containing 5% skim milk in Tris-buffered saline with 0.1% Tween 20 (TBS-T) for 2 h at room temperature and then incubated with primary antibodies against AKT (1:1000 dilution, #4691, Cell Signaling Technology), p-AKT (1:2000 dilution, #4060, Cell Signaling Technology), PI3K (1:1000 dilution, ab191606, Abcam), p-PI3K (1:1000 dilution, ab278545, Abcam), eNOS (1:1000 dilution, #32027, Cell Signaling Technology), p-eNOS (1:1000 dilution, ab215717, Abcam), and Mas (1:1000 dilution, #85003-1-RR, Proteintech) overnight at 4°C with shaking. The membrane was washed with TBS-T for 15 min and then incubated with horseradish peroxidase (HRP)–conjugated goat anti-rabbit secondary antibody (Wuhan Sanying Biotechnology Co., Ltd.) at a 1:2000 dilution in TBS-T and 3% skim milk for 90 min at room temperature. The membrane was washed three times with TBS-T, each for 15 min, and the immune reaction signals were detected using an ECL detection method. Protein bands were detected using a chemiluminescent gel imaging system (Bio-Rad Lab, China). Protein expression was quantitatively analyzed using ImageJ software (National Institutes of Health, Bethesda, MD, United States), and the experiment was repeated three times.

### 2.18. Application of MYR-DM-ANG1-7 in Diabetic Mouse Skin Infection Model

BALB/c male mice are from the Experimental Animal Center of Air Force Military Medical University. One group was fed a normal diet, and the other five groups were fed a high-fat diet for 4 weeks and then injected with 35 mg/kg streptozotocin (STZ, 35 mg/kg, Sigma) intraperitoneally for 3 days. The measured fasting blood glucose levels of mice were all greater than 16.7 mmol/L; that is, the model was successfully established. After intraperitoneal injection of pentobarbital sodium (1%, 0.3 mg/kg), the dorsal hair of each animal was shaved off, and a circular full-thickness skin wound was created on the back using a 10-mm sterile mouse punch. *S. aureus* (ATCC 25923) was used to infect the wound site of mice, and a full-thickness wound infection model of mice was established. *S. aureus* was first resuscitated by operation in a biological ultra-clean table. A single colony was picked and incubated in LB medium at 37°C and 200 rpm. The concentration of the above *S. aureus* solution was adjusted to 1 × 10^8^ CFU/mL to infect the wound of mice. Then, 24 h after *S. aureus* infection, three drugs, DM (20 *μ*g/mL), ANG1-7 (20 *μ*g/mL), and MYR-DM-ANG1-7 (20 *μ*g/mL), were dissolved in PBS buffer, and 200 *μ*L was injected subcutaneously for 3 days. After drug injection, the wound pus was diluted and coated with plate culture to observe the colony situation. The gross specimen imaging system (GF-7A, China) was used to record the wounds of mice on the same day and every other day after the formation of the wounds. The rubber ring with adhesive backing was used to fix the wound and prevent it from shrinking. ImageJ analysis software (NIH, Bethesda, MD, United States) was used to calculate and analyze the wound area. After 0, 3, 7, 10, and 14 days, the size of the infected wound was evaluated by rubber ring. The wound closure was measured as follows: the wound healing rate (%) = [*W*_0_ − *W*_*n*_]/*W*_0_ × 100%, where *W*_0_ and *W*_*n*_ represent the wound area on the 0th and *n*th days, respectively (*n* = 4).

### 2.19. Histological Evaluation

On the 14th day, the skin tissue was fixed in 4% paraformaldehyde solution, dehydrated, embedded in paraffin, sliced, and stained with hematoxylin–eosin (HE) and Masson. Skin tissue sections on Day 7 were immunofluorescently stained using rabbit anti-mouse CD31 (Cell Signaling Technology) and *α*-SMA (Cell Signaling Technology), and all stained sections were observed under the slide scanner (Aperio AT 2; Leica Biosystems, Germany).

### 2.20. Statistical Analysis

Statistical differences were determined by one-way ANOVA and unpaired *T*-test using GraphPad Prism 8. A value of *p* < 0.05 is considered significant. All data are shown as mean ± standard deviation (SD).

## 3. Result

### 3.1. Characterization of MYR-DM-ANG1-7

The purity and molecular weight of the peptide were confirmed by HPLC (Figure S1) and mass spectrometry (Figure S2), respectively. Figure S1 showed a single main peak at 11.707 min in the HPLC chromatogram of AMPs, and the purity was more than 95%. The mass spectrum results of Figure S2 showed that the main peak m/z 5428.08 was consistent with the theoretical molecular weight of the target peptide (5428.38 Da), which verified its molecular structure.

**Figure 1:**
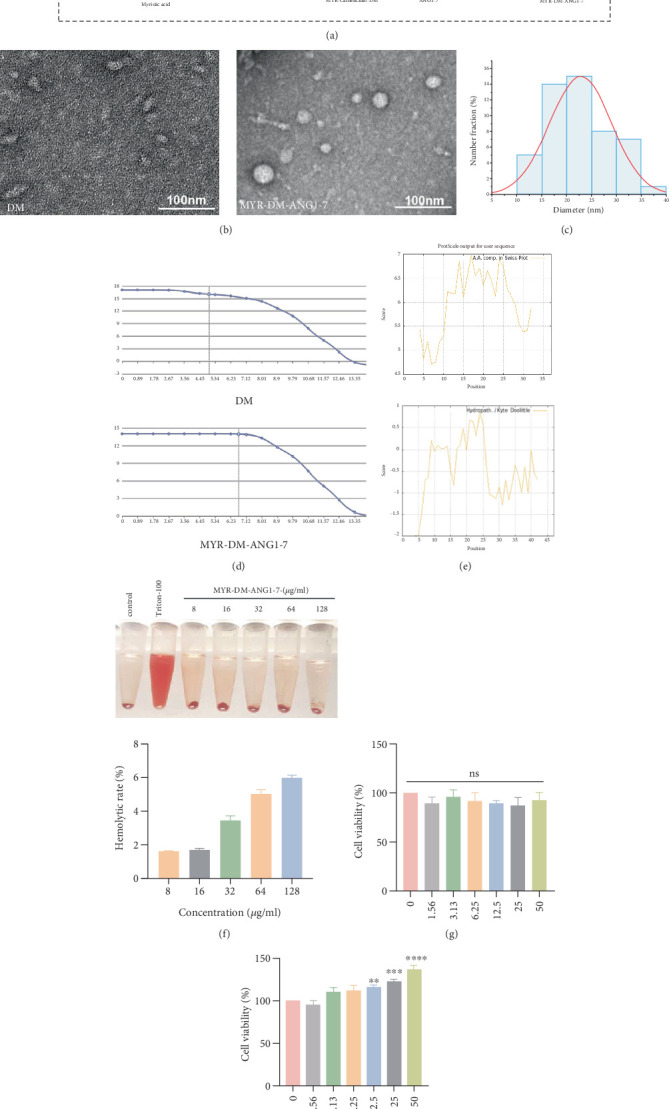
Structural characterization of MYR-DM-ANG1-7. (a) Reasonable engineering was achieved by introducing myristoylation at the N-terminal of DM and sequential nanoassembly of ANG1-7 at the C-terminal. (b, c) Transmission electron microscope (TEM) images of cathelicidin-DM and MYR-DM-ANG1-7 dispersed nanoparticles in ultrapure water. Scale bar represents 100 nm. (d, e) Analysis of the net charge and hydrophobicity of MYR-DM-ANG1-7 and DM. (f) Qualitative and quantitative analysis of hemolytic activity of MYR-DM-ANG1-7. (g) Analysis of HaCaT cell viability. (h) Analysis of HSF cell viability.

DM and MYR-DM-ANG1-7 (32 *μ*g/mL) were characterized by TEM. The results showed that (Figure 1b) after modification, MYR-DM-ANG1-7 novel AMPs could spontaneously form critical micelle structures in deionized water, and the particle size was 22.38 ± 1.63 nm.

The net charge and hydrophobicity of antibacterial peptides are very important for their antibacterial effect and cell selectivity. Net charge affects the electrostatic interaction between AMPs and bacterial membranes, while hydrophobicity determines its ability to penetrate cell membranes. Proper balance of net charge and hydrophobicity can enhance antibacterial activity and reduce toxicity to host cells. As shown in Figure 1d,e, firstly, we predict and analyze the net charge and hydrophobicity of the peptide sequence. Compared with the parent peptide, the net charge of MYR-DM-ANG1-7 increases by two, which can improve the corresponding antibacterial ability. Then, the hydrophobic prediction of the polypeptide sequence without myristic acid showed that the sequence had obvious hydrophilic characteristics. However, when myristic acid is introduced into the sequence, an amphiphilic structure with one hydrophobic end and one hydrophilic end is formed, and the optimization of this structure significantly enhances the ability of the polypeptide to self-assemble into micelles.

In order to evaluate the safety of the drug in vitro, we took blood from rat eyeball to determine the hemolytic activity of the drug, and the results are shown in Figure 1f. The hemolysis rate of self-assembled peptides increased with the increase of concentration. The hemolysis rate of red blood cells was 6.01% at the concentration of 128 *μ*g/mL, but it was less than 5% at the concentration of 8–64 *μ*g/mL, which showed low hemolysis and cytotoxicity and high biological safety.

Then, we conducted cytotoxicity tests on HSFs and HaCaTs, which are very important for wound healing. The cells were treated with different concentrations of MYR-DM-ANG1-7, and the cell viability was evaluated by CCK8 test. As shown in Figure 1g,h, the survival rate of HSF cells treated with MYR-DM-ANG1-7 increased in a concentration-dependent manner. At the lowest concentration (1.56 *μ*g/mL), the cell survival rate was 95.16%, and with the increase of concentration, the survival rate gradually increased. This indicates that MYR-DM-ANG1-7 not only has no obvious cytotoxicity but also may promote the survival of HSF cells. At the same time, MYR-DM-ANG1-7 showed excellent biocompatibility in HaCaTs, and the survival rate was above 90% at all tested concentrations, and there was no significant difference among different concentrations. Even at the highest concentration (50 *μ*g/mL), the cell survival rate remained at 92.4%.

### 3.2. MYR-DM-ANG1-7 Has Antibacterial Activity Against *S. aureus* and *E. coli* and Biofilm Resistance

**Figure 2:**
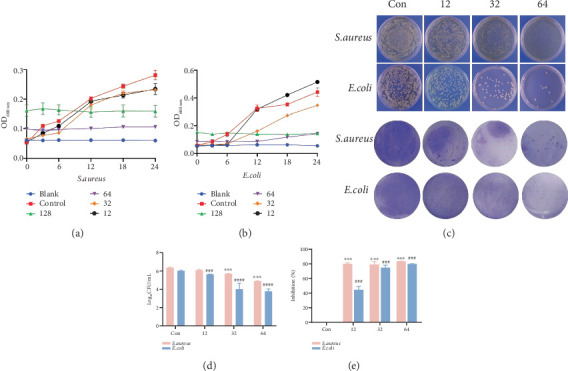
Antimicrobial and antibiofilm activities of MYR-DM-ANG1-7. (a, b) Inhibition curves of MYR-DM-ANG1-7. (c) Digital images of biofilms stained with crystal violet (CV) and the live bacterial status within the biofilms. (d) Quantitative analysis of CV-stained biofilms. (e) Quantitative analysis of live bacteria within the biofilms. The levels of statistical significance between the control and treated samples are indicated as follows: ⁣^∗∗^*p* < 0.01 and ⁣^∗∗∗^*p* < 0.001 vs. Con. ^###^*p* < 0.001,^#####^*p* < 0.0001, and #*p* < 0.001 vs. Con.

*S. aureus* and *E. coli* are the most commonly isolated pathogens in diabetic wound infection. Their infection rate is high in diabetic patients, especially in the case of poor blood sugar control, so the above strains are selected as representative model microorganisms for antibacterial tests. The antibacterial effect of MYR-DM-ANG1-7 was evaluated (Table 1 and Figure 2). Firstly, the minimal inhibitory concentration (MIC) of MYR-DM-ANG1-7 was 16 *μ*g/mL for *S. aureus* and 12 *μ*g/mL for *E. coli*. After that, the concentrations of 1 × MIC, 2 × MIC, 3 × MIC, and 4 × MIC AMPs were cocultured with bacteria in the logarithmic growth period, and the OD value was measured at 600 nm. As shown in Figure 2a,b, AMPs at a concentration of 4 × MIC (128 *μ*g/mL) can completely inhibit the growth of *E. coli* and *S. aureus*.

The formation of bacterial biofilms in the wounds of DFU patients is the main cause of bacterial resistance [26]. By measuring the antibiofilm ability, the inhibitory and clearing effects of drugs on biofilms can be assessed. In the case of preventing biofilm formation, *E. coli* and *S. aureus* were cultured in LB nutrient medium with different concentrations of the novel AMP MYR-DM-ANG1-7 in 24-well plates at 37°C and incubate for 24 h. CV staining can observe the remaining amount of *E. coli* and *S. aureus* biofilms treated with different concentrations of drugs. As shown in Figure 2, the untreated control showed a dark purple color, indicating more biofilm biomass, and the MYR-DM-ANG1-7-treated group showed a significantly lighter color, indicating a better biofilm inhibition ability. The viable bacterial count results (Figure 2) showed that the number of viable bacteria in the biofilm of the experimental group was significantly reduced. The experimental data (Figure 2) also show that the MYR-DM-ANG1-7 self-assembled nanostructure can effectively inhibit the formation of *E. coli* and *S. aureus* biofilms.

### 3.3. MYR-DM-ANG1-7 Can Improve the Proliferation and Migration Ability of HUVECs Treated With HG + LPS

**Figure 3:**
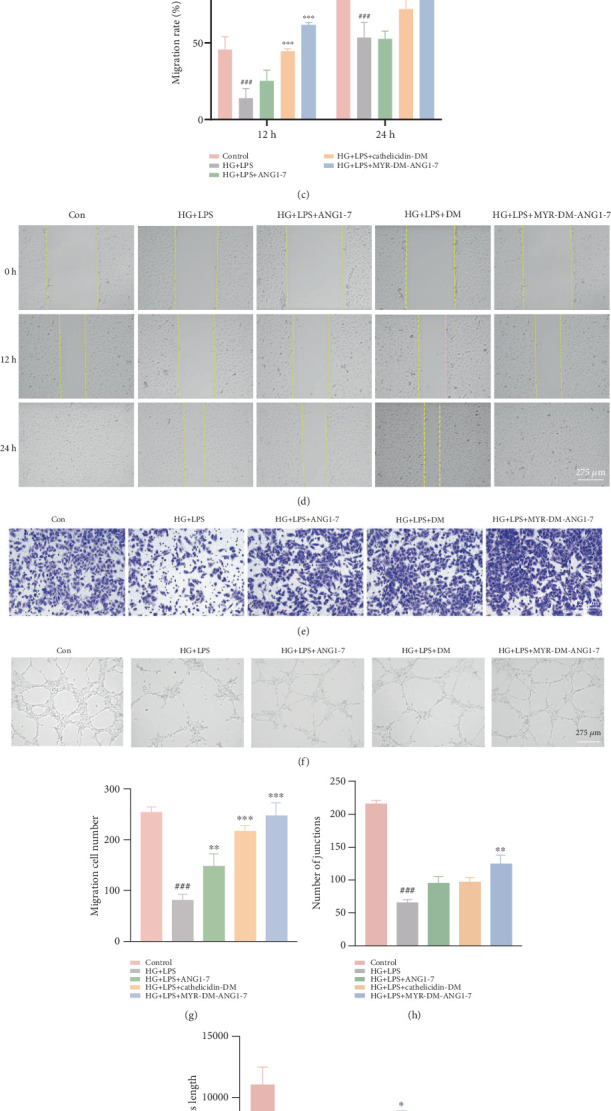
MYR-DM-ANG1-7 promotes endothelial cell proliferation, migration, and angiogenesis. (a) HUVECs treated with 35 mmol HG and different concentrations of LPS (0.1, 1, 10, 100, and 1000 ng/mL) for 24 h. (b) HUVECs treated with different concentrations of MYR-DM-ANG1-7 for 24 h. Cell viability was measured using the CCK-8 assay. (c) Semiquantitative measurement of migration area using ImageJ in the in vitro wound healing assay, and calculation of cell migration rate in the scratch area. (d) Migration of HUVECs stimulated by different drug treatment groups using 20 *μ*g/mL ANG1-7, cathelicidin-DM, and MYR-DM-ANG1-7, with a scale bar representing 275 *μ*m. (e) Transwell migration assay to detect endothelial cell migration. (f) The ability of ANG1-7, MYR-DM-ANG1-7, and DM treatment groups to stimulate HUVECs to induce tube formation in vitro. Representative cell images show the field of view. (g) Quantitative migration data using ImageJ. (h, i) Semiquantitative analysis of branch nodes and length of formed vessels using ImageJ. Data are represented as mean ± standard deviation (SD) (*n* = 3). Statistical analysis: ⁣^∗^*p* < 0.05, ⁣^∗∗^*p* < 0.01, and ⁣^∗∗∗^*p* < 0.001 vs. HG + LPS. ^###^*p* < 0.001 vs. Con.

HUVECs were treated with 35 mmol/L glucose and different concentrations of LPS (0.1, 1, 10, 100, and 1000 ng/mL) for 24 h, and it was found that HG + LPS induced cytotoxicity (Figure 3a). Therefore, the concentration of 35 mmol HG + 100 ng/mL LPS was considered to be a suitable concentration for the following experiments. In order to detect the cytoprotective effect of MYR-DM-ANG1-7 on HG-induced HUVEC cytotoxicity, cells were treated with different doses of MYR-DM-ANG1-7 (2, 5, 10, and 20 *μ*g/mL). As shown in Figure 3b, exposure of HUVEC to a concentration of 35 mmol HG + 100 ng/mL LPS for 24 h induced cytotoxicity, and the cell viability was 53.68% (*p* ≤ 0.001). However, the cytotoxic effect of HG on HUVECs could be significantly inhibited after MYR-DM-ANG1-7 pretreated cells, and the viability of cells treated with 20 *μ*g/mL drug was 81.91% (*p* < 0.01).

We used cell scratch assay to study the effects of different drugs on cell migration of HUVECs. The results shown in Figure 3d showed that the migration rate of endothelial cells was attenuated by HG + LPS and restored after stimulation with MYR-DM-ANG1-7. Further analysis of migration ability by indirectly measuring the remaining space in the wound and calculating the ratio (Figure 3c) confirmed the results observed in Figure 3d. After 12 h of action, the wound healing rates of the control group, HG group, ANG1-7 group, DM group, and MYR-DM-ANG1-7 group were 45.7%, 13.8.%, 44.7%, 25.2%, and 61.6%, respectively. At 48 h, the healing area of the control group and MYR-DM-ANG1-7 group had reached 100%, while the healing area of the ANG1-7 group and DM group only increased to 72.06% and 52.6%. Transwell migration is shown in Figure 3e,g. The results show that compared with the control group, the number of cells cultured in HG + LPS passing through the chamber was significantly reduced (*p* < 0.01). At the same time, the number of cells stimulated by the new AMP drug increased as the number of cells passing through the chamber increased (*p* < 0.01).

Based on the above endothelial cell results, the vascularization ability of the novel AMP in the HG + LPS environment was explored through tube formation experiments. As shown in Figure 3f, endothelial cells in the control group exhibited better and more tube formation compared with cells treated with HG + LPS to simulate the inflammatory and oxidative environment of diabetes. In Figure 3h,i, the main connections and length of the blood vessel-like structures formed in the HG + LPS group were significantly reduced to 0.3 times and 0.4 times, respectively. The endothelial cells in the MYR-DM-ANG1-7 group showed stronger blood vessel formation ability; the main connections and length of the formed blood vessels increased, respectively, to 1.9 times and 1.7 times, while the main connections and lengths of blood vessels formed by the remaining two groups ANG1-7 and DM increased to 1.4 times and 1.3 times, respectively. More main connections and lengths indicate better formation of the vascular network; this is necessary for wound healing.

### 3.4. MYR-DM-ANG1-7 Regulates PI3K/AKT-eNOS Signaling Pathway In Vitro

**Figure 4:**
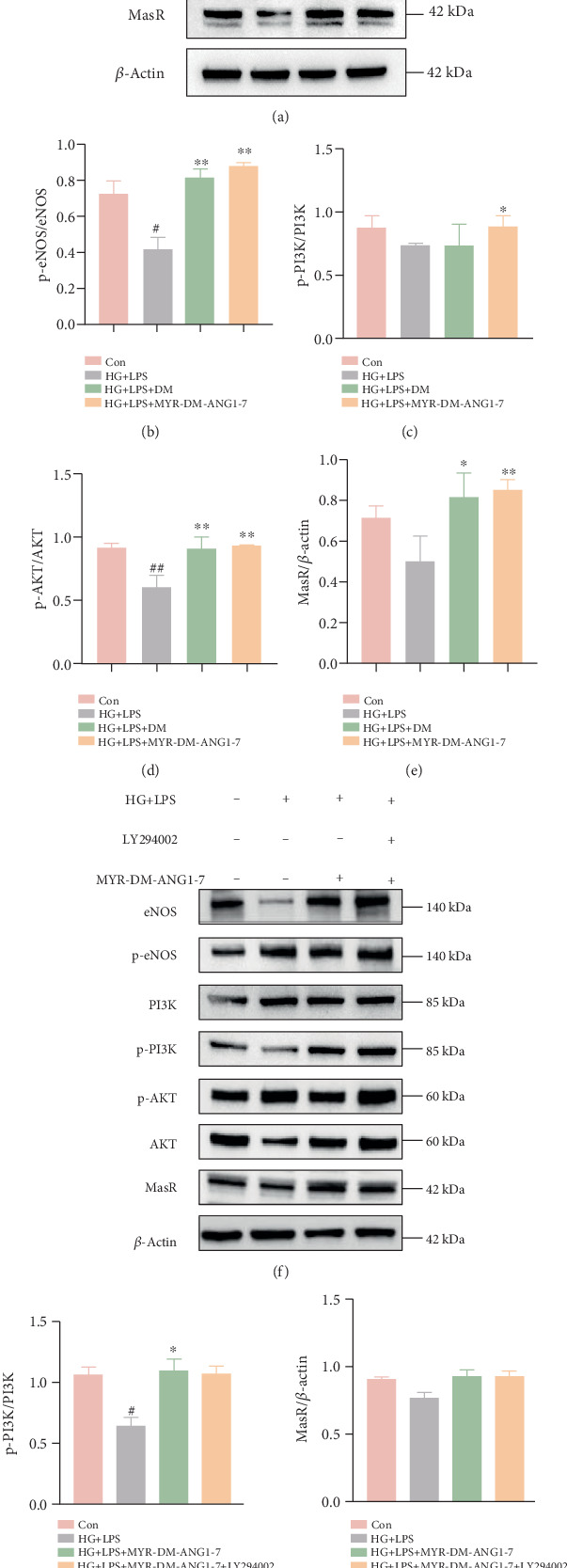
MYR-DM-ANG1-7 participates in vascular regulation through the PI3K/AKT/eNOS signaling pathway. (a) Western blot detection of the expression of total AKT, phosphorylated AKT, total PI3K, phosphorylated AKT, total eNOS, phosphorylated eNOS, and MasR proteins in endothelial cells of the control group, HG + LPS group, DM group, and MYR-DM-ANG1-7 group. (b–e) Semiquantitative analysis of the phosphorylation levels of p-eNOS/total eNOS, p-AKT/total AKT, and p-PI3K/total PI3K. (f) Detection of related protein expression after the addition of the inhibitor LY294002. (g–j) Semiquantitative analysis of the phosphorylation levels of p-eNOS/total eNOS, p-AKT/total AKT, and p-PI3K/total PI3K. Data are represented as mean ± SD (*n* = 3). Statistical analysis: ⁣^∗^*p* < 0.05, ⁣^∗∗^*p* < 0.01, and ⁣^∗∗∗^*p* < 0.001 vs. HG + LPS. ^##^*p* < 0.01 vs. Con.

**Figure 5:**
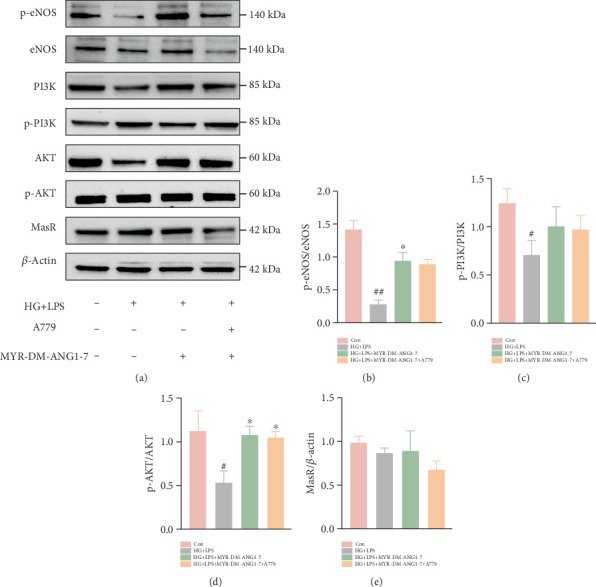
MYR-DM-ANG1-7 regulates the PI3K/AKT/eNOS signaling pathway through MasR to participate in vascular regulation. (a) Western blot detection of related protein expression after the addition of the inhibitor A779. Detection of the expression of total AKT, phosphorylated AKT, total PI3K total eNOS, phosphorylated eNOS, and MasR proteins in endothelial cells of the control group, HG + LPS group, cathelicidin-DM group, and MYR-DM-ANG1-7 group. (b–d) Semiquantitative analysis of the phosphorylation levels of p-eNOS/total eNOS, p-AKT/total AKT, and p-PI3K/total PI3K. (e) Semiquantitative analysis of MasR protein expression. Data are represented as mean ± SD (*n* = 3). Statistical analysis: ⁣^∗^*p* < 0.05, ⁣^∗∗^*p* < 0.01, ⁣^∗∗∗^*p* < 0.001 vs. the HG + LPS group. ^##^*p* < 0.01 and ^#^*p* < 0.05 vs. the control.

Previous studies have shown that the PI3K/AKT/eNOS signaling pathway is involved in the protective effect on endothelial cell dysfunction and apoptosis [27]. In the PI3K/AKT/eNOS signaling pathway, PI3K, AKT, and eNOS are key proteins in endothelial dysfunction in diabetes. They are not only involved in metabolic disorders of diabetes but also are closely related to diabetes-related vascular complications. In our study, the expression and phosphorylation levels of key proteins AKT, PI3K, and eNOS were evaluated (Figure 4). The ratio of phosphorylated protein to total protein was further calculated to represent the phosphorylation level of the corresponding protein. These Figures 4, 4, 4, and 4 show that HG + LPS stimulation inhibited the phosphorylation levels of AKT, PI3K, and eNOS (downregulated from 0.9-fold to 0.4-fold and 0.9-fold to 0.8-fold). After MYR-DM-ANG1-7 pretreatment,the phosphorylation level of AKT was increased (from 0.4-fold to 0.94-fold), the phosphorylation level of PI3K was increased (from 0.8-fold to 1.28-fold), and the phosphorylation level of eNOS was increased. We then added the inhibitor LY294002, and Western blot analysis showed that the increase in p-PI3K, p-AKT, and p-eNOS levels was significantly inhibited in the inhibitor group. However, MYR-DM-ANG1-7 pretreatment increased the phosphorylation levels of AKT, PI3K, and eNOS (Figure 4, 4, 4, 4, and 4). We also found that the expression of the membrane receptor MasR was reduced after HG treatment, and MasR was significantly increased after drug treatment. This effect may be mediated through the MasR. Inhibitor A779 (MasR inhibitor) was subsequently added for further verification. After the inhibitor was added (Figure 5), the expression of MasR protein was reduced, and the phosphorylation levels of AKT and PI3K were weakened, indicating that the PI3K/AKT/eNOS pathway can be indirectly inhibited by inhibiting the expression of MasR. MYR-DM-ANG1-7 treatment can increase the number of endothelial cells, sourced from ammonia monoxide synthase (eNOS) phosphorylation site Ser 1177. The phosphorylation levels, as well as the expression levels of p-PI3K and p-AKT, suggest that we can restore the PI3K/AKT/eNOS pathway effect after pretreating cells with MYR-DM-ANG1-7.

### 3.5. MYR-DM-ANG1-7 Reduces the Oxidative Stress in HUVECs Induced by HG + LPS

**Figure 6:**
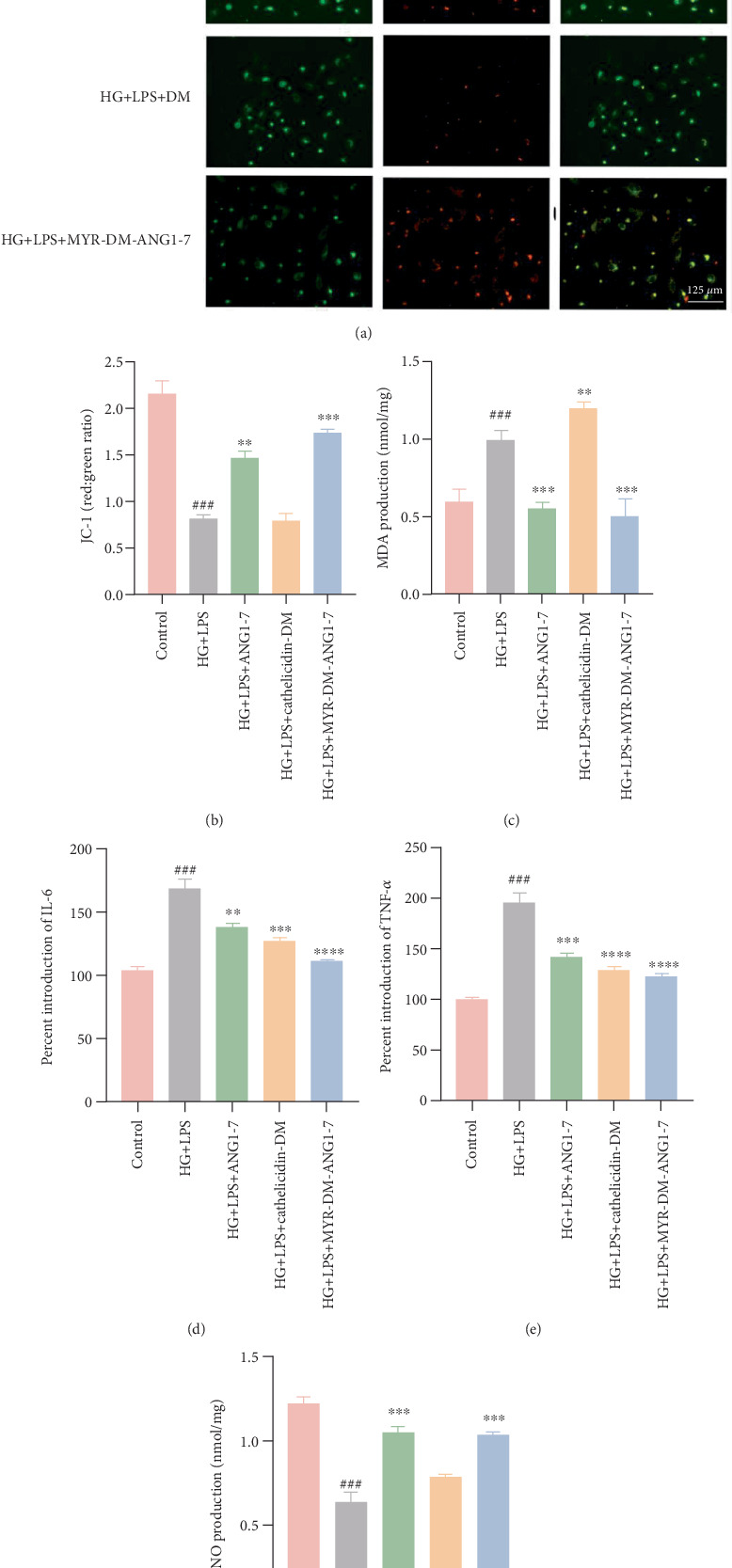
MYR-DM-ANG1-7 modulates the production of oxidants, antioxidants, and inflammatory mediators induced by HG + LPS. (a, b) Fluorescence microscopy examination of endothelial cells and further semiquantitative analysis of intracellular mitochondrial membrane potential JC-1 in the control group, HG + LPS group, ANG1-7 group, MYR-DM-ANG1-7 group, and DM group. (c) NO secretion in endothelial cells of the control group, HG + LPS group, ANG1-7 group, MYR-DM-ANG1-7 group, and DM group. (d) Detection of MDA in the supernatant of endothelial cells in the control group, HG + LPS group, ANG1-7 group, MYR-DM-ANG1-7 group, and DM group. (e, f) Cytokine expression of IL-6 in endothelial cells of the control group, HG + LPS group, and other groups measured by enzyme-linked immunosorbent assay (ELISA). Data are represented as mean ± SD (*n* = 3). Statistical analysis: ⁣^∗∗^*p* < 0.01 and ⁣^∗∗∗^*p* < 0.001 vs. the HG + LPS group. ^###^*p* < 0.001 vs. the control.

In patients with diabetes, the energy metabolism function of mitochondria will be impaired in a high blood sugar environment. Therefore, we evaluated the effects of ANG1-7, MYR-DM-ANG1-7, and DM on MMP (JC-1) in endothelial cells exposed to HG + LPS. As shown in Figure 6a,b, compared with the control group, the MMP in endothelial cells incubated in the HG + LPS group was reduced and green fluorescence was produced (*p* < 0.0001). Treating cells with a concentration of 20 *μ*g/mL can increase their membrane potential (*p* < 0.0001), reducing mitochondrial damage, indicating that the modified peptide can protect HUVEC from HG + LPS-induced mitochondrial damage. In addition, MYR-DM-ANG1-7 (20 *μ*g/mL) reduced the intracellular reactive oxygen species level by approximately 32% (compared with the HG + LPS group, *p* < 0.01), which was consistent with the decrease in MMP observed in Figure S3.

MDA is considered an indicator of lipid peroxidation in patients with diabetes [28]. Further investigation of the oxidant MDA is also shown in Figure 6c, which is related to oxidative stress. Under the stimulation of HG + LPS, the secretion of MDA increased significantly by 1.65 times. After treatment with MYR-DM-ANG1-7, the secretion of MDA decreased by 0.51 times. The secretion of MDA in the other two groups after treatment with ANG1-7 significantly decreased by 0.56. The amount of MDA secretion increased by 1.2 times after treatment with DM.

We further examined the expression of two key proinflammatory cytokines, including IL-6 and TNF-*α*. As shown in Figure 6d,e, compared with the negative control group, the secretion of these proinflammatory cytokines was significantly increased after HG + LPS treatment of HUVEC for 24 h (*p* < 0.0001). In addition, MYR-DM-ANG1-7 stimulation can alleviate the decrease in IL-6 and TNF-*α* levels in HUVECs induced by HG + LPS.

### 3.6. MYR-DM-ANG1-7 Stimulates NO Secretion in HUVECs Induced by HG + LPS

In the diabetic state, increased oxidative stress leads to decreased eNOS activity in vascular endothelial cells [29]. Since NO is synthesized by eNOS, its reduced bioavailability results in impaired vascular relaxation function. Therefore, decreased NO synthesis is one of the main factors contributing to endothelium-dependent vascular relaxation dysfunction in diabetes [30]. Next, we assess the impact of drugs on NO synthesis by quantitatively analyzing NO secretion levels. In Figure 6f, when endothelial cells are stimulated with HG + LPS to mimic an inflammatory and oxidative environment, NO secretion decreases to 0.5 times the original level. After treatment with MYR-DM-ANG1-7, NO production in the cell supernatant increases to 1.63 times. The NO secretion in the other two groups treated with ANG1-7 significantly rises to 1.65 times, and in the group treated with DM, NO secretion significantly increases to 1.2 times.

### 3.7. MYR-DM-ANG1-7 Can Promote the Healing of Diabetic Infectious Wounds in Mice, Reduce Bacterial Colonization, and Significantly Improve Wound Neovascularization

**Figure 7:**
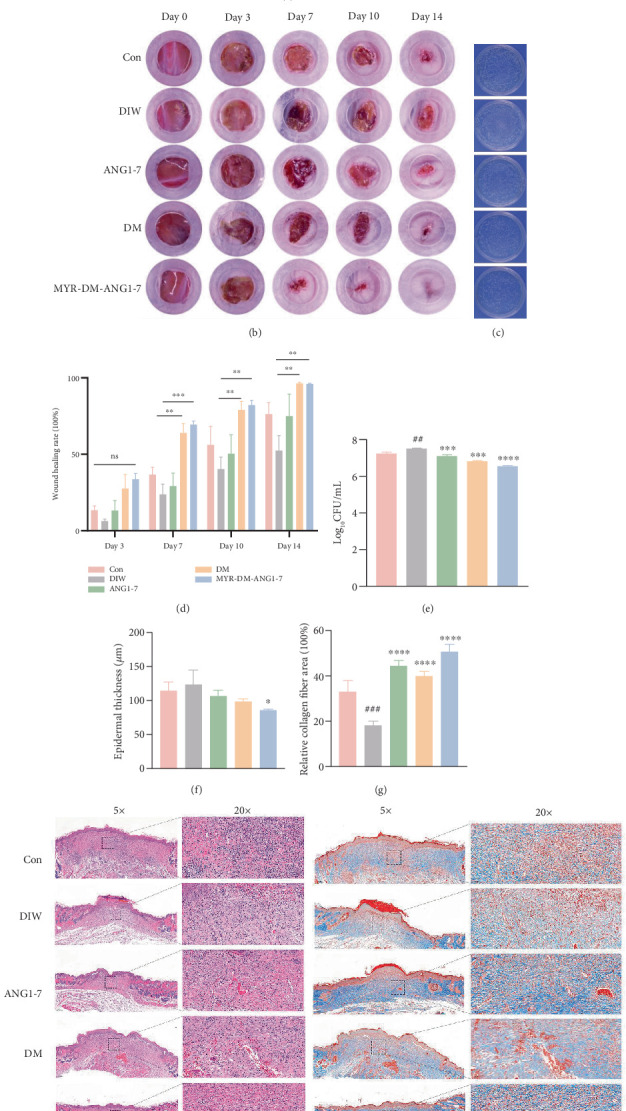
MYR-DM-ANG1-7 promotes the healing of diabetic infected wounds. (a) Schematic diagram of the construction of diabetic infected wounds. (b) Photographs of wounds at different time points (on the 0th, 3rd, 7th, 10th, and 14th days from injection) in normal infected mice, diabetic infected mice, and diabetic mice treated with ANG1-7, DM, and MYR-DM-ANG1-7. (c) Observation of bacterial colonies in wound exudate after drug injection. (d) Calculation and comparison of wound healing area percentage at different time points (on the 3rd, 7th, 10th, and 14th days from injection) for each group. (e) Quantitative analysis of bacterial colonies. (f) Analysis of relative collagen fiber density in wound tissue on Day 14. (g) Analysis of epidermal thickness in wound tissue on Day 14. (h) HE and Masson staining of wound tissue on Day 14 (scale bar represents 200 *μ*m, 5×; scale bar represents 50 *μ*m, 20×). Data are represented as mean ± SD (*n* = 4). Statistical analysis: ⁣^∗^*p* < 0.05, ⁣^∗∗^*p* < 0.01, and ⁣^∗∗∗^*p* < 0.001.

The results in Figure 7 show that in the diabetic animal model, the wound healing ability is weakened throughout the healing process. The wound healing area in the diabetic control group was significantly smaller than that in the normal group. On the third day, the wound healing rate in the ordinary group was 13.33%, while the healing rate in the diabetic control group was only 6.32%. On the 7th day, the three groups of indicators of ANG 1-7, DM, and MYR-DM-ANG1-7 were 40.69%, 69.86%, and 72.77%, respectively. Compared with the diabetic control group, the wound surface in the MYR-DM-ANG1-7 treatment group was significantly reduced (*p* < 0.001). In the late stage of wound healing, in each group of treatments, the corresponding percentages in the diabetic control group were 40.26% on the 10th day and 52.34% on the 14th day. The corresponding healing rates of the MYR-DM-ANG1-7 treatment group reached 85.22% and 95.66% on the 10th and 14th days, respectively. The infected wound area of diabetic mice treated with MYR-DM-ANG1-7 was smaller than that of the diabetic control group (*p* < 0.01). After 24 h of treating the wound with drugs, as shown in Figure 7, the pus from the wound was diluted and incubated on an LB agar plate to count bacterial colonies. It was found that the number of bacteria in the wound treated with MYR-DM-ANG1-7 decreased (*p* < 0.0001).

On Day 14, skin wound sections were subjected to HE staining and Masson's trichrome staining. As shown in Figures 7f, 7g, and 7h, MYR-DM-ANG1-7 promotes tissue re-epithelialization in mice and granulation tissue formation, while the wound length is less than that treated with ordinary infection. On the 14th day after trauma, compared with the diabetes infection group, the mice in the MYR-DM-ANG1-7 group still had more wound granulation tissue than the control group, and the epidermis became thinner and transformed into normal tissue. In Masson staining, the collagen deposition area in the MYR-DM-ANG1-7 group became larger and the collagen fibers became denser (*p* < 0.0001), which indicated that more collagen was generated during the healing process, which is a sign of good healing effect.

**Figure 8:**
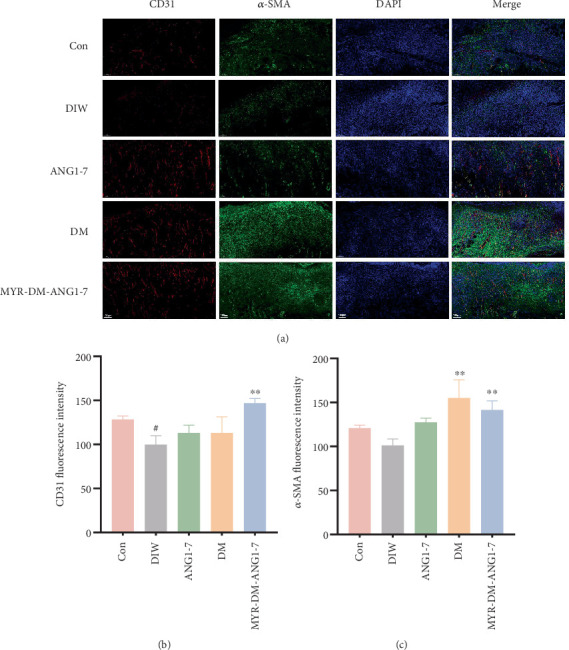
MYR-DM-ANG1-7 promotes angiogenesis in diabetic infected wound tissue. (a) CD31 and *α*-SMA immunofluorescence staining of wound tissue on Day 7. (b, c) Quantitative analysis of CD31 and *α*-SMA fluorescence. Data are represented as mean ± SD (*n* = 3). Statistical analysis: ^#^*p* < 0.05 vs. the control. ⁣^∗∗^*p* < 0.01 vs. diabetes infection.

However, in the diabetic environment, the formation of new blood vessels in the wound is weakened [31]. To explore whether MYR-DM-ANG1-7 can promote the formation of new blood vessels, we used CD31 (one of the specific surface molecules of endothelial cells) and *α*-smooth muscle actin (*α*-SMA) to evaluate new blood vessels. In Figure 8, CD31 immunofluorescence staining and further analysis showed that more blood vessels could be found in diabetic wounds treated with MYR-DM-ANG1-7 compared with the diabetes infection group (*p* < 0.05). Additionally, *α*-SMA was used to quantify the maturation of newly generated blood vessels. On the seventh day, the fluorescence intensity of *α*-SMA in the DM and MYR-DM-ANG1-7 treatment groups was significantly higher than that in the diabetes infection group (*p* < 0.01). In short, MYR-DM-ANG1-7 can increase the nutrient supply to tissues around the wound by promoting angiogenesis and subsequently accelerate collagen deposition.

Finally, we evaluated the in vivo biocompatibility of the self-assembling peptide MYR-DM-ANG1-7 by measuring and observing the pathological changes in the liver and kidneys of different groups on the 14th day. Our research results indicated that no evidence of organ damage or pathological changes was found in the group using MYR-DM-ANG1-7 (Figure S4). These results demonstrated that the self-assembling peptide MYR-DM-ANG1-7 exhibited excellent biocompatibility.

## 4. Discussion

The chronic nonhealing nature of diabetic wounds stems from a vicious cycle of polymicrobial infections and impaired angiogenesis. Polymicrobial infections are critical contributors to diabetic wound recalcitrance, with wounds frequently colonized by diverse Gram-positive or Gram-negative bacterial strains. These bacteria exacerbate antibiotic resistance through biofilm formation [32]. Concurrently, vascular dysfunction—a hallmark of diabetic wounds—impairs oxygen and nutrient transport during tissue repair, leading to delayed healing [8, 33]. Therefore, there is an urgent need for therapeutic strategies that integrate antibacterial and proangiogenic functions to treat diabetic-infected wounds and reduce amputation-related morbidity and mortality. In this study, the modularly engineered peptide MYR-DM-ANG1-7 was developed to combine antimicrobial sensitization and vascular endothelial repair.

AMPs, known for their broad-spectrum antibacterial activity and diverse wound-healing-promoting mechanisms, are promising candidates for topical antibiotics [34, 35]. However, natural AMPs exhibit insufficient stability in clinical applications due to protease susceptibility and reduced bioavailability caused by nonspecific immune responses in wounds [32, 36]. Through screening AMP databases, we identified DM, an amphibian-derived *α*-helical peptide with activity against both Gram-positive and Gram-negative bacteria. Although previous studies confirmed DM's bactericidal effects and ability to promote HUVEC proliferation and migration, its antibiofilm properties, mechanisms underlying promigratory effects, and in vivo efficacy in diabetic-infected wound models remained unexplored.

Fatty acyl modifications with 6–14 carbon chains enhance AMPs' antibacterial activity, reduce immunogenicity, and critically improve protease resistance by sterically shielding cleavage sites [37, 38]. For example, chensinin-1b (an *α*-helical AMP) forms pH-responsive supramolecular micelles after N-terminal hexanoic acid modification, wherein the hydrophobic core physically blocks protease access [39]. Inspired by this, we introduced a myristoyl group (14-carbon chain) at the N-terminus of DM and conjugated ANG1-7 to its C-terminus via a GGG flexible linker. The GGG linker effectively reduces the spatial hindrance between functional domains while maintaining the conformational flexibility required for receptor binding [40]. This design strategy is similar to the clinically validated approach in semaglutide, where the *γ*Glu-2xOEG-*γ*Glu linker not only optimizes the spatial interval between the fatty acid chain and the receptor binding domain but also enhances the interaction with serum albumin, thereby improving pharmacokinetic properties without compromising biological activity. This design achieves excellent protease resistance and enhanced antibacterial activity through the synergistic effect of dual mechanisms. The tetradecanoyl moiety is embedded in the hydrophobic region, spatially blocking the proteolytic cleavage site and forming an N-terminal hydrophobic shield, preventing recognition by exopeptidases. Meanwhile, it self-assembles into monodisperse nanostructures, generating a micelle-mediated protective effect—the dense hydrophobic core repels the aqueous phase enzymes due to the low dielectric environment, blocking protease binding. The synergy of molecular-level steric hindrance and nanoscale isolation significantly enhances biological stability while maintaining therapeutic function. Additionally, the optimized hydrophobicity and net charge (+ 7 vs. DM + 5) further enhance the antibacterial activity. ANG1-7 further endowed the peptide with targeted vascular repair by binding MasRs on endothelial cells. MYR-DM-ANG1-7 self-assembled into micelles (22.38-nm diameter at 32 *μ*g/mL), driven by hydrophobic interactions (myristoyl core) and hydrogen/electrostatic bonding (ANG1-7 shell) [41]. Importantly, MYR-DM-ANG1-7 exhibited negligible cytotoxicity toward endothelial cells, fibroblasts, or keratinocytes and minimal hemolytic activity.

CV staining and MIC assays confirmed that MYR-DM-ANG1-7 significantly enhanced antibiofilm activity and antibacterial potency against *S. aureus* (Gram-positive) and *E. coli* (Gram-negative), with MIC values lower than those of the parent peptide DM. The antibacterial efficacy of MYR-DM-ANG1-7 increased 1.3-fold compared to DM, attributed to structural optimization through myristoylation. The micellar surface, enriched with cationic ANG1-7 (+ 7 net charge), strengthened electrostatic adsorption to anionic bacterial membranes. The GGG linker ensured covalent conjugation of ANG1-7 to DM without disrupting its *α*-helical structure, simultaneously increasing net charge and conferring MasR-targeted proangiogenic activity.

In a *S. aureus*-infected diabetic mouse model, MYR-DM-ANG1-7 demonstrated spatiotemporal synergy between early-phase antibacterial action and late-phase tissue repair. At Day 3 posttreatment, MYR-DM-ANG1-7 reduced bacterial colonization more effectively than DM, likely due to micelle-mediated drug retention via steric hindrance, which protected the peptide from proteolytic degradation and prolonged its presence in wound exudate. By Day 14, histological analysis (HE and Masson staining) revealed restored epidermal thickness, increased collagen density, and organized collagen deposition in MYR-DM-ANG1-7-treated wounds. Immunofluorescence confirmed enhanced CD31+/*α*-SMA+ neovascularization, directly linking the peptide's activity to vascular regeneration.

Using an HG + LPS-induced HUVEC injury model, MYR-DM-ANG1-7 outperformed DM or ANG1-7 alone in promoting proliferation, migration, and tube formation [42]. Mechanistically, MYR-DM-ANG1-7 preserved MMP, reduced MDA levels, and elevated NO production—critical for maintaining vascular homeostasis [43, 44]. However, whether ANG1-7 can repair HUVECs induced by HG + LPS through the Mas/PI3K/AKT/eNOS signaling pathway has not been reported [45]. This design addressed the lack of vascular targeting in the parent peptide DM. Activation of the PI3K/AKT/eNOS pathway via ANG1-7-Mas receptor binding was central to these effects: ANG1-7 triggered PI3K phosphorylation, leading to AKT activation and eNOS-dependent NO synthesis. These findings were validated using the PI3K inhibitor LY294002 and the MasR antagonist A779, which abolished the protective effects.

In conclusion, MYR-DM-ANG1-7, engineered through modular design, represents a dual-functional peptide integrating antimicrobial and proangiogenic activities via a “bacterial clearance–anti-inflammation–repair” cascade. Myristoylation, an FDA-approved modification (e.g., in daptomycin), ensures translational safety, while ANG1-7 leverages endogenous signaling to avoid immunogenicity. Nevertheless, the micellar release kinetics, immunomodulatory effects (e.g., macrophage polarization toward M2 phenotype), and long-term resistance risks remain uncharacterized. Future studies should employ fluorescence tracing, single-cell sequencing, and metagenomics to address these limitations.

## Figures and Tables

**Table 1:** Antimicrobial activity of MYR-DM-ANG1-7 and DM.

**Microorganism**	**Minimum inhibitory concentration (*μ*g/mL)**	
	**DM**	**MYR-DM-ANG1-7**
ATCC25922	16	12
ATCC25923	16	12

## Data Availability

The data that support the findings of this study are available from the corresponding author upon reasonable request.

## Supporting Information

Supporting Information Additional supporting information can be found online in the Supporting Information section. Figure S1: Analysis of HPLC chromatogram results of antimicrobial peptides. Figure S2: Analysis of MS results of antimicrobial peptides. Figure S3: Flow cytometry was used to show the ROS fluorescence in different treatment groups. Figure S4: Representative H&E-stained images of liver and kidney tissues in each group (scale bar: 200 *μ*m).
