# Supplementary material for: Myristoylated Cathelicidin-DM Fused With ANG1-7: A Novel Self-Assembling Antimicrobial Peptide for the Treatment and Mechanism of Diabetic Infected Wounds
Source: J Diabetes Res. 2025 Sep 2;2025:9601959. doi: 10.1155/jdr/9601959 (PMC12419922; doi:10.1155/jdr/9601959)
Supplement: Supporting Information — Additional supporting information can be found online in the Supporting Information section. Figure S1: Analysis of HPLC chromatogram results of antimicrobial peptides. Figure S2: Analysis of MS results of antimicrobial peptides. Figure S3: Flow cytometry was used to show the ROS fluorescence in different treatment groups. Figure S4: Representative H&E-stained images of liver and kidney tissues in each group (scale bar: 200 μm). [file 9601959.f1.docx]

| 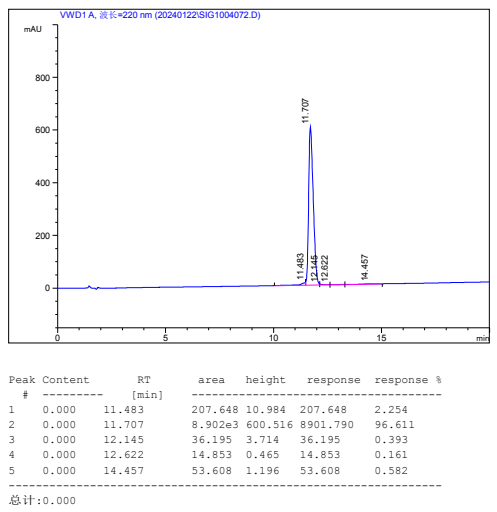 |
| --- |
| **Figure S1** **Analysis of HPLC Chromatogram Results of Antimicrobial Peptides**   \|  \| MS Spectrum \| \| --- \| --- \| \| 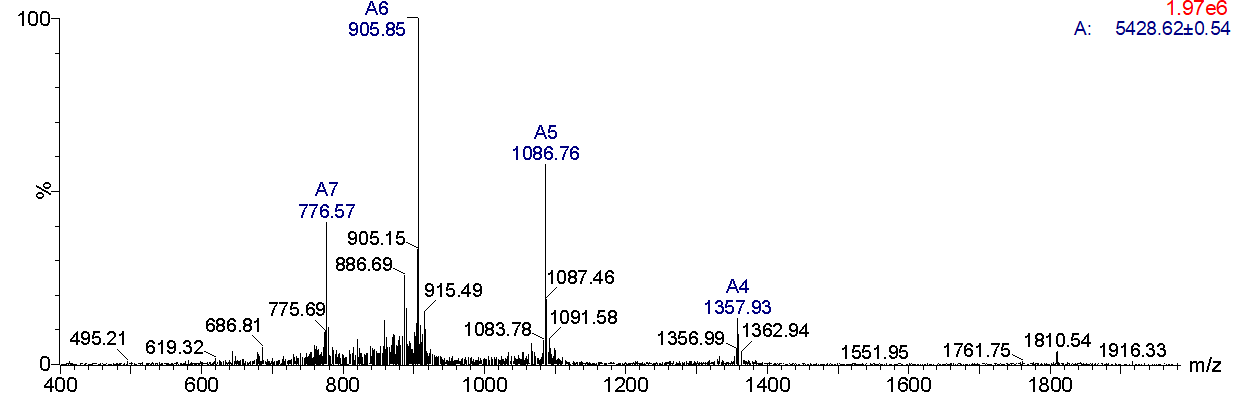 \| \| \| **Figure S2** **Analysis of MS Results of Antimicrobial Peptides** \| \|  \|  \| |

| 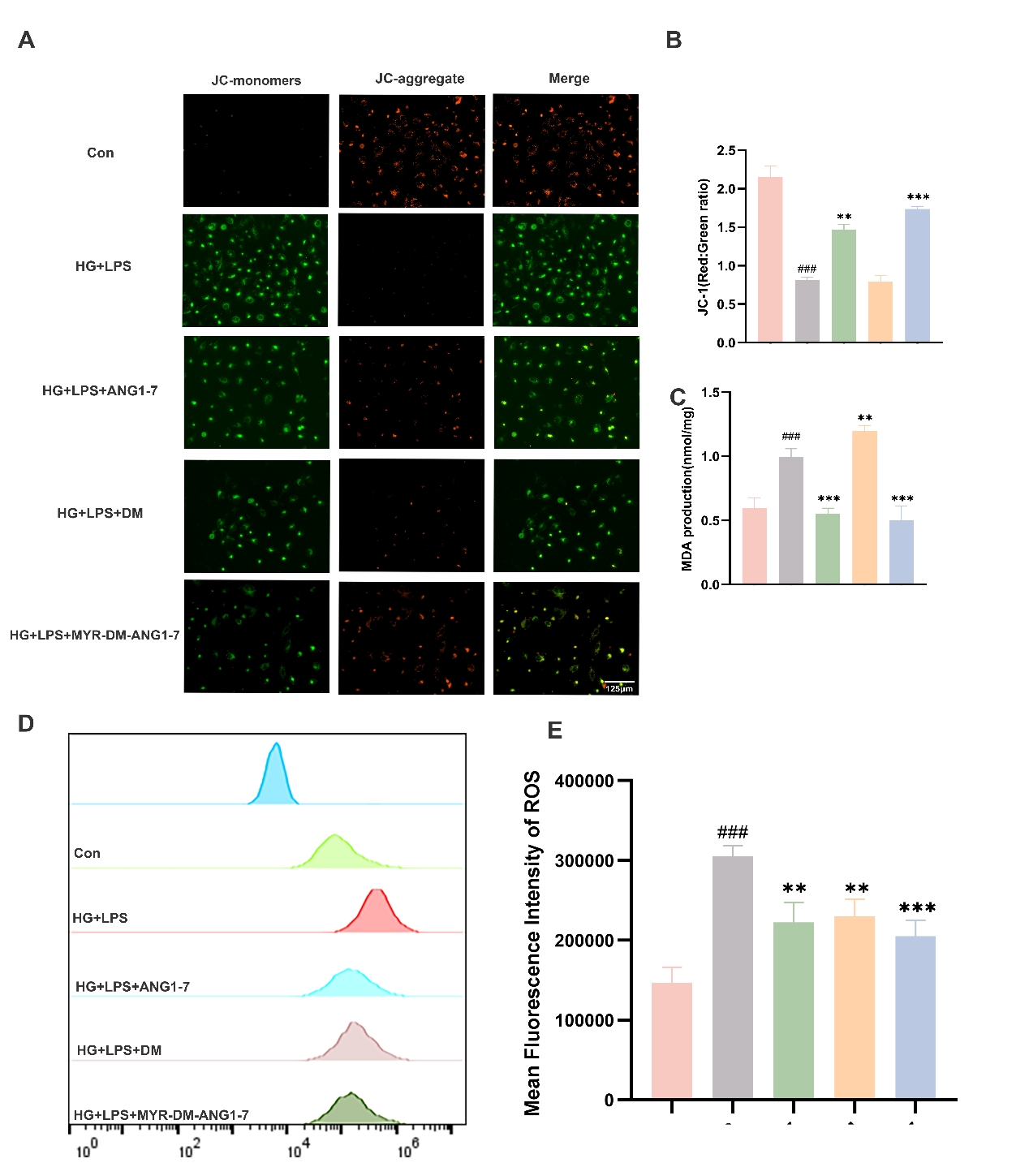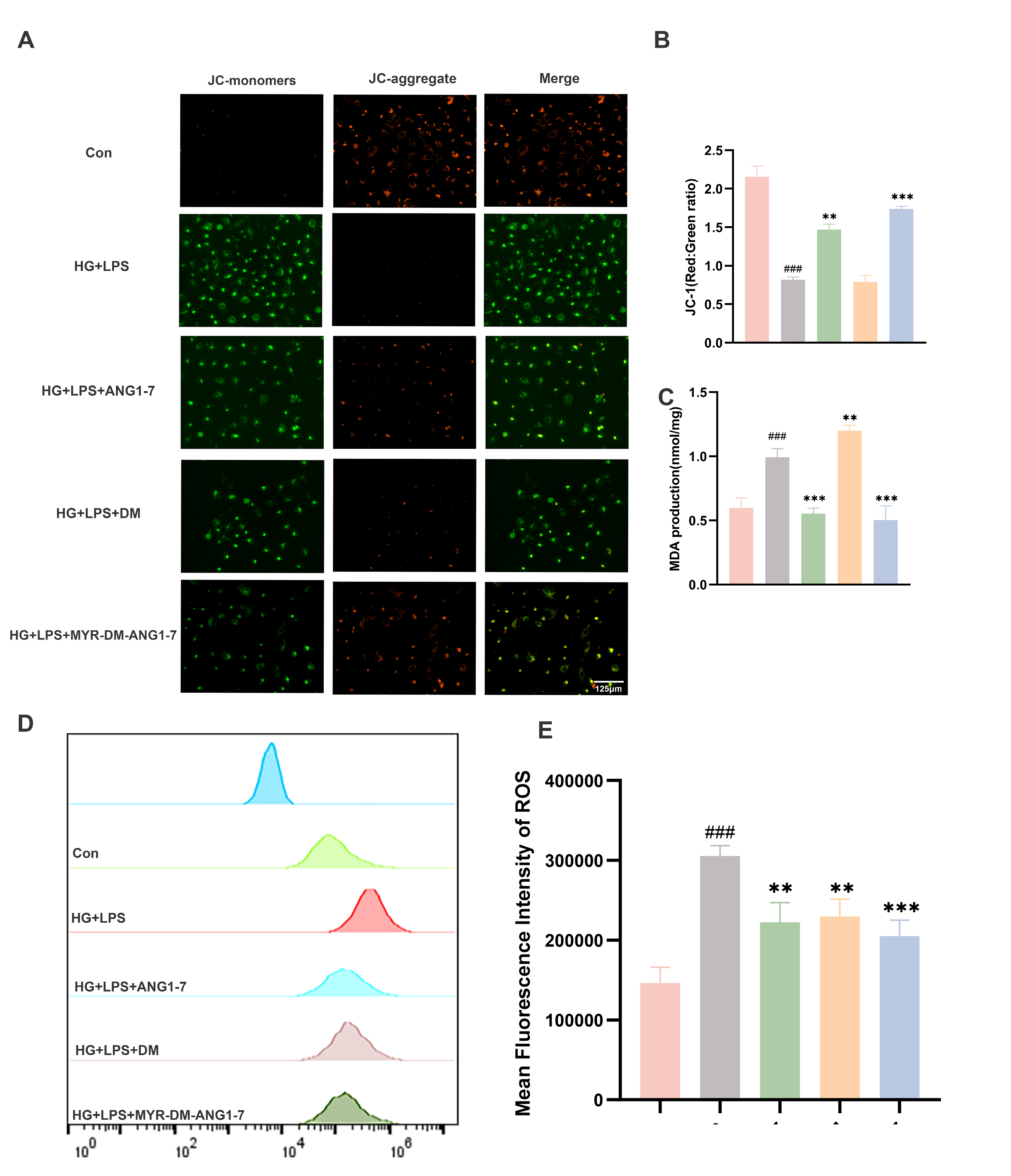 |
| --- |
| **Figure S3** **Flow cytometry was used to show the ROS fluorescence in different treatment groups.** |

| 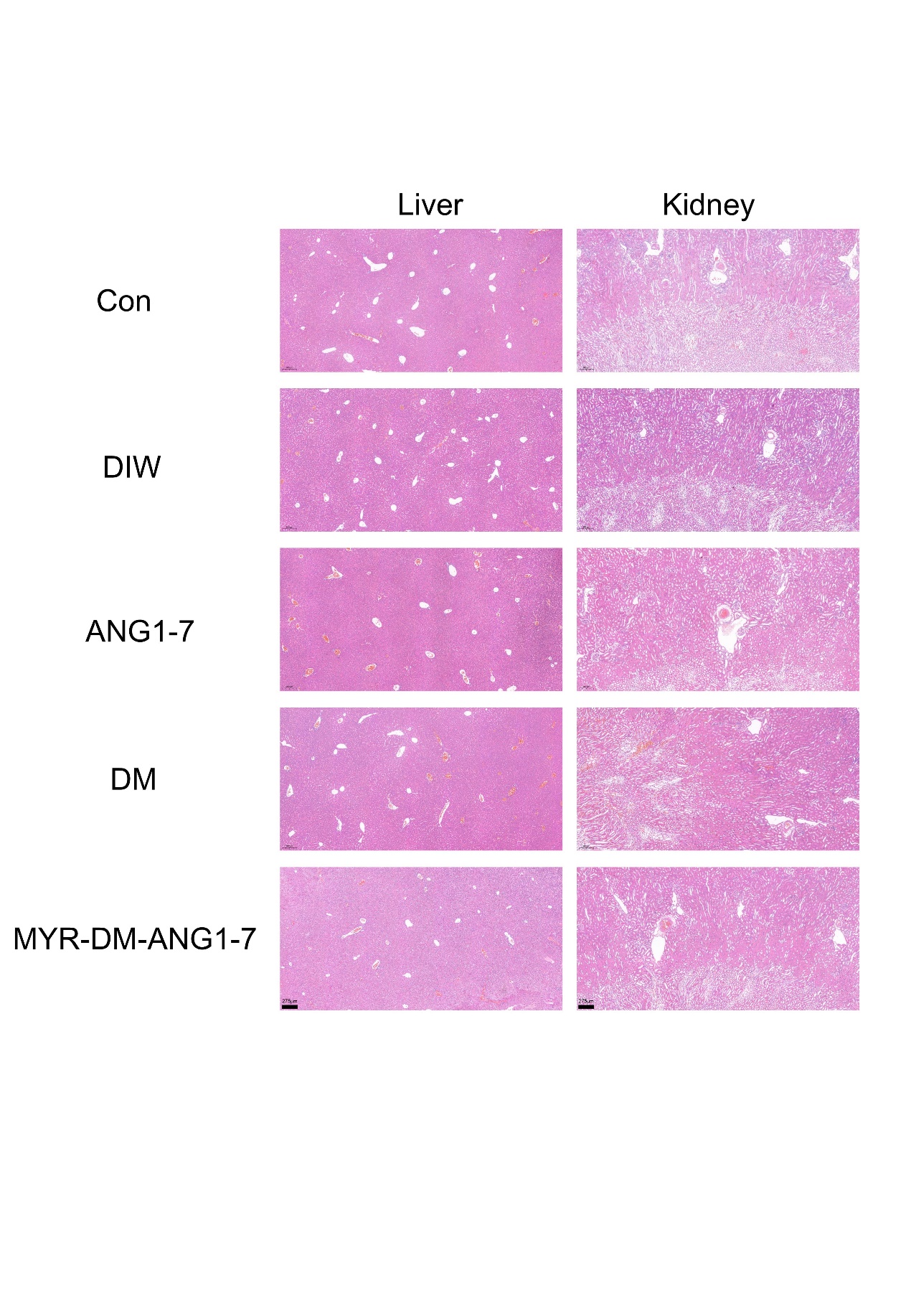 |
| --- |
| **Figure S4. Representative H&E stained images of liver and kidney tissues in each group (scale bar: 200 μm).** |
